# Visceral Obesity, Metabolic Syndrome, and Esophageal Adenocarcinoma

**DOI:** 10.3389/fonc.2021.627270

**Published:** 2021-03-12

**Authors:** Jessie A. Elliott, John V. Reynolds

**Affiliations:** Trinity St. James's Cancer Institute, Trinity College Dublin and St. James's Hospital, Dublin, Ireland

**Keywords:** esophageal cancer, adenocarcinoma, obesity, visceral obesity, metabolic syndrome, Barrett's esophagus, gastroesophageal reflux, tumor microenvironment

## Abstract

Esophageal adenocarcinoma (EAC) represents an exemplar of obesity-associated carcinogenesis, with a progressive increase in EAC risk with increased body mass index. In this context, there is increased focus on visceral adipose tissue and associated metabolic dysfunction, including hypertension, diabetes mellitus and hyperlipidemia, or combinations of these in the metabolic syndrome. Visceral obesity (VO) may promote EAC *via* both directly impacting on gastro-esophageal reflux disease and Barrett's esophagus, as well as *via* reflux-independent effects, involving adipokines, growth factors, insulin resistance, and the microbiome. In this review these pathways are explored, including the impact of VO on the tumor microenvironment, and on cancer outcomes. The current evidence-based literature regarding the role of dietary, lifestyle, pharmacologic and surgical interventions to modulate the risk of EAC is explored.

## Introduction and Background

The past four decades have seen a marked rise in the incidence of esophageal adenocarcinoma (EAC), with data from the United States demonstrating a continued rise in the overall incidence from ~0.4 per 100,000 in 1975 to 3.5 per 100,000 in 2015—an over 8-fold increase (1). These reports occur against a background increase in the worldwide prevalence of obesity between 1975 and 2014 of 3.6-fold for males and 2.3-fold for females, with some 640 million adults, and 110 million children, exhibiting obesity globally (2). If these trends continue, it is predicted by the year 2025, the worldwide prevalence of obesity will rise to ~18% for males and over 21% for females, with Class 3 obesity in 6% for males and 9% for females (2).

EAC is an aggressive cancer and has traditionally been associated with poor oncologic outcomes. However, prognosis for patients treated with curative intent have improved, with a combination of treatments, including preoperative chemotherapy and radiation therapy followed by surgery resulting in the landmark CROSS trial in a 47% 5-year overall (3). Encouragingly, for all patients, the recent SURVMARK data indicate that 5-year survival from esophageal cancer almost doubled in high income countries when the period 1995–99 is compared with 2010–14, with a survival increase from 11 to ~22% (4–7). A further major advance in esophageal cancer is an increased identification of patients with early mucosal cancer, and its management by minimally invasive endotherapy approaches. A major current focus consequently is both on an understanding of the pathophysiology of carcinogenesis, and on further improvements in therapies to continue recent improved trends.

In this context, the link between obesity and EAC is of major interest. The incidence rates have parallels, and moreover epidemiologic studies have established a clear association between obesity and the risk of EAC (8–14). For instance, the International Agency for Research on Cancer (IARC) recently reported a progressive increase in relative risk for EAC per 5 kg/m^2^ increase in BMI, suggesting a dose-response effect (15). Two pathways may be relevant. First, visceral obesity (VO) may promote GERD which if severe or protracted is a risk factor for EAC (16, 17). Second, intriguingly, *via* GERD-independent effects, common to obesity-associated cancers such as pancreatic, uterine, colorectal, renal, liver, and postmenopausal breast, including altered glucose and lipid metabolism, and the elaboration by VAT of cytokines and growth factors, and the promotion of systemic inflammation. VO moreover is associated with metabolic dysregulation and inflammation which links to predisposition to type 2 diabetes mellitus (T2DM), non-alcoholic fatty liver disease (NAFLD), and cardiovascular disease (18). An emerging research focus is how obesity-associated inflammation may impact on the key hallmarks of cancer within the tumor microenvironment (TME) (19, 20).

Metabolic syndrome (MetS) defines a distinct clinical phenotype that is associated with VO, with added features of T2DM, hypertension and hyperlipidemia, and commonly NAFLD (21, 22). Patients with MetS may also exhibit micro-albuminuria, endothelial dysfunction and a pro-inflammatory and pro-thrombotic state, with increased circulating C-reactive protein (CRP), tumor necrosis factor-α (TNF-α) and interleukin-6 (IL-6). MetS is associated with increased risk of several cancer types, in particular hepatocellular cancer (HCC), however it is also associated with EAC possibly *via* the pathologic precursor of Barrett's esophagus (BE) (23).

Notwithstanding a clear epidemiologic association, the impact of VO and MetS on oncologic outcomes of EAC remains uncertain, with various data suggesting improved, worsened or an unchanged survival and treatment outcomes (24–27). With respect to esophageal cancer surgery, although VO may impact operative outcomes through increased technical difficulty, and associated comorbidities may increase perioperative risk, the current literature are highly inconsistent in this regard (26), possibly due to the differential impact of various stages of obesity and comorbidity on perioperative risk, and the confounding effect of malnutrition and loss of lean body mass, known as sarcopenia, which is present in many patients with esophageal cancer (28, 29). Across oncology a greater understanding of obesity and tumor biology is sought, and a 2014 statement from the American Society of Clinical Oncology (ASCO) commits to addressing this issue through increased education, and the delivery resources for healthcare professionals to tackle obesity in partnership with patients, in addition to increasing provisions for public health policy supporting the prevention and treatment of obesity (30).

The present review aims to summarize key data outlining the roles of obesity, VO, and the MetS in the pathophysiology of EAC, with a focus on potential targets for prevention and treatment.

## Epidemiologic Links

### Obesity and EAC

A strong and consistent relationship between EAC risk and obesity has been reported in a number of large, prospective, population-based studies. At present the largest pooled study assessing the relationship between EAC risk and obesity includes individual patient data from 12 population-based studies including almost 4,000 patients with EAC and adenocarcinoma of the esophagogastric junction (AEG), and over 10,000 control subjects. This reported a dose-response effect of BMI with the greatest risk among individuals with the most severe obesity (OR = 4.76 [95% CI: 2.96–7.66] for class III obesity [BMI ≥ 40 kg/m^2^]; OR = 2.79 [95% CI: 1.89–4.12] for class II obesity [BMI: 35–39.9 kg/m^2^]; OR = 2.39 [95% CI: 1.86–3.06] for class I obesity [BMI: 30–34.9 kg/m^2^]; and OR = 1.54 [95% CI: 1.26–1.88] for overweight [BMI: 25–29.9 kg/m^2^], vs. BMI <25 kg/m^2^) (8).

In the National Institutes of Health American Association of Retired Persons cohort, BMI and waist-hip ratio (WHR, a measure of central obesity) were associated with increased EAC risk on multivariable analysis (31). Notably, even for normal weight individuals, WHR was associated with an increased risk. The most current systematic review and meta-analysis of VO as a risk factor for esophageal cancer included seven studies up to August 2016 including 913,182 patients (32). Higher waist circumference (WC) and WHR were associated with greater risk of esophageal cancer (WC: RR = 2.06, 95% CI: 1.30–3.24; WHR: RR = 1.99, 95% CI: 1.05–3.75).

### Metabolic Syndrome and EAC

MetS defines VO in association with metabolic consequences such as T2DM and hyperlipidemia, and typically is associated with a pro-inflammatory systemic and local response, and end-organ consequences such as NAFLD. Studies have identified an association between MetS and both BE and EAC. A systematic review and meta-analysis of MetS and BE identified 12 publications including 355,311 subjects and reported a significant association (OR = 1.23; 95% CI: 1.03–1.47). For EAC, with MetS defined using a standardized metabolic risk score, a significant association between the metabolic risk score and EAC was observed exclusively in male patients (33). Analysis of the SEER dataset also demonstrated that EAC was significantly associated with MetS (OR = 1.16; 95% CI: 1.06–1.26) vs. control subjects. Interestingly, among males, the association between EAC and MetS appeared independent of prior GERD (34).

### GERD, Obesity, and EAC

A key relevant pathway linking obesity with EAC is *via* GERD, as long duration or severe GERD is associated with an up to 40-fold increase risk of EAC (35). There is an approximate 3-fold risk of GERD among patients with obesity compared with normal weight individuals (36), and a doubling of the risk of erosive esophagitis (37). VO in particular is associated with increased distal esophageal acid exposure and increased prevalence of hiatus hernia (38). Despite the clear association between obesity and GERD, a large pooled study which examined the relationship between obesity, GERD and risk of EAC, found that the size and direction of the association between BMI and EAC was not significantly different among patients with and without GERD, highlighting the role of reflux-independent mechanisms in obesity-associated esophageal carcinogenesis (39). There did appear however to be a synergy between obesity and GERD with respect to risk of EAC, as the risk of cancer among persons with GERD was significantly greater than predicted using an additive statistical model.

BE is a recognized precursor to EAC, and the current consensus is that chronic acid and bile refluxate in the distal esophagus leads to esophagitis, and triggers a metaplasia to dysplasia to EAC sequence (40). Individuals with obesity are at increased risk for the development of BE, with increased visceral adiposity a key driver *via* greater GERD severity (35, 41). VO and MetS are more commonly seen among patients with BE, with the strongest relationship observed among those with long segment BE, suggesting a possible role for VO in the pathogenesis of BE (42). At meta-analysis, patients with VO were at increased risk of developing BE (aOR = 1.98, 95%CI: 1.52–2.57) (39), and in five studies this persisted even after adjustment for BMI (43–46). In addition, among eleven studies wherein patients with GERD served as controls, or adjustment for symptomatic GERD was undertaken, a reflux-independent association was observed between BE and VO (aOR = 2.04, 95%CI: 1.44–2.90) (41, 43, 47–55). These data strongly indicate that the increased incidence of BE among patients with VO occurs through both GERD-dependent and -independent mechanisms, with the presence of MetS an added risk factor.

## Key Pathways and Mediators Linking Obesity, Metabolic Syndrome and EAC

### Acid and Bile, Local Inflammation, and Carcinogenesis

At a cellular level, it appears that chronic exposure to gastric refluxate initiates an inflammatory process within the esophageal squamous epithelium, eventually leading to metaplasia and dysplasia. Gastric acid, a principal component of gastric refluxate, may directly influence the development and progression of BE (56, 57). However, similar distal esophageal acid exposure has been demonstrated among patients erosive esophagitis as compared with BE, suggesting that additional factors may impact the mutational burden seen in BE and EAC (57).

The impact of bile on esophageal carcinogenesis is of interest, in particular in view of the association of BE with bile reflux, and the epidemiologic link with VO. In rodent models, surgically-induced bile reflux or gavage of bile into the esophagus produces severe esophagitis, BE and EAC (58–60). This produces increases in key local inflammatory mediators such as IL-6, IL-8, and COX-2 (61, 62), resulting in oxidative stress and DNA damage, leading to genomic instability and carcinogenesis (58, 62, 63). *In vitro* studies demonstrate that esophageal squamous epithelial cells exposed to bile acids exhibit altered gene expression profiles, leading to intestinal metaplasia. The effects of bile acids on esophageal squamous epithelium appear to be pH dependent suggesting a synergistic effect between gastric acid and bile with respect to the development and progression of BE to EAC (58, 64, 65).

**Figure 1:**
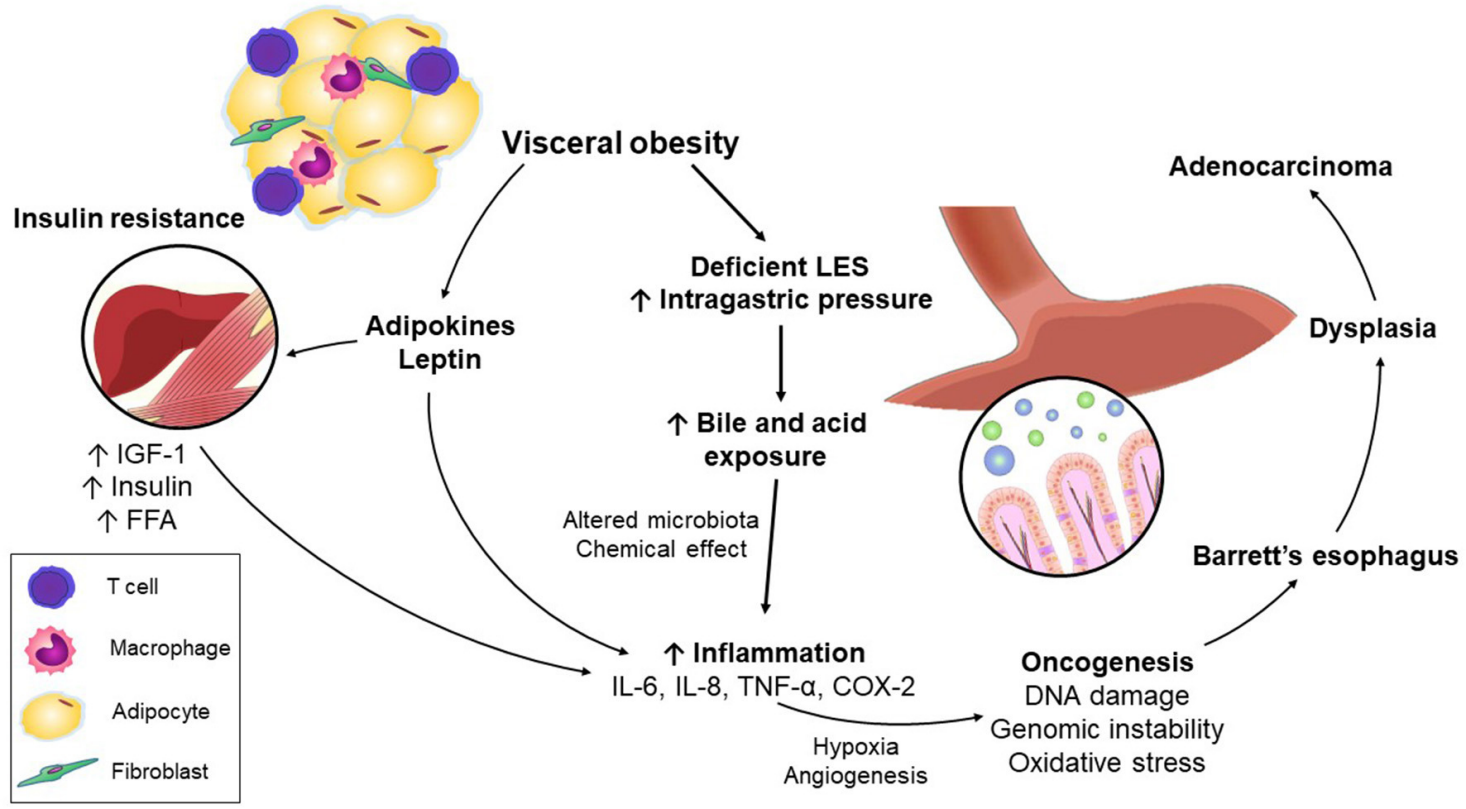
Mechanisms linking visceral obesity and esophageal adenocarcinoma.

Esophageal injury by acid and bile may result in mutational changes in BE, irrespective of grade of dysplasia (66–68). Aneuploidy and driver gene mutations develop early in the pathogenesis of EAC and are evident even among patients with non-dysplastic BE. Killcoyne et al. (69) demonstrated that using whole-genome sequencing from patients in BE surveillance over up to 15 years, genomic signals could distinguish between progressive and stable disease long before the development of dysplasia, highlighting the importance of accumulated DNA damage and genomic instability in the progression of BE to EAC (Figure 1).

### Systemic Inflammation

Adipose tissue comprises two main depots—subcutaneous and visceral (70). Visceral adipose tissue (VAT), including the omentum, mesentery, epiploic, epicardial, gonadal, and retroperitoneal fat deposits, contains increased pro-inflammatory macrophages and T cells, and growth factors, relative to subcutaneous fat, and is a key driver of inflammation and altered metabolism in obesity (71). The “portal hypothesis” proposes that the relatively increased contribution of VAT to metabolic dysfunction and end-organ damage may relate to its drainage *via* the portal vein to the liver, with consequent increased hepatic exposure to free fatty acids (FFA) and pro-inflammatory mediators, with potential consequences including NAFLD and insulin resistance (72). Notwithstanding, the mechanisms involved in initiation of adipose tissue inflammation are not completely understood.

Other proposed pathways include lipotoxicity and consequent endoplasmic reticulum stress, toll-like receptor activation and impaired oxygenation (73). Inflamed VAT may also contribute pro-inflammatory cytokines. IL-6 excess may activate STAT3, while increased TNF-α is associated with induction of *c-myc* oncogene expression (74–76). In a study of patients with BE, increased IL-6 and C-reactive protein (CRP) was associated with progression to HGD/EAC (77). While *in vitro* studies have demonstrated that culture of EAC cells with VAT or in adipose conditioned medium results in up-regulation of markers of proliferation, invasion and metastasis such as MMP-2 and−9, and attenuates tumor suppressor p53 (78, 79), obesity is also associated with increased markers of epithelial to mesenchymal transition in EAC, highlighting the role of adipose-secreted factors in the pathogenesis of EAC in obesity (80).

### Adipokines

In addition to its central role as an energy store, VAT also serves as an endocrine organ, producing adipokines, bioactive molecules which have both metabolic and immune-regulatory functions (Table 1). Leptin was first identified in 1994 as the *obese* (ob) gene product in mice, and we now understand that leptin serves an important role as an adipostasis hormone. Leptin is secreted by white adipocytes, and exists in the circulation at levels proportionate to body fat mass (81–83). In rodent models, exogenous leptin acts on the hypothalamus to reduce calorie intake and increase energy expenditure (84). The key functional importance of leptin in body weight homeostasis is highlighted by the obese and hyperphagic *ob/ob* mouse which lacks leptin, and the *fa/fa* Zucker diabetic fatty rat which exhibits a leptin receptor mutation (85). Leptin deficiency and mutations in the leptin receptor are recognized rare causes of monogenic early onset, severe obesity (86, 87), and recombinant leptin replacement therapy produces significant clinical benefit in these patients (88).

**Table 1:** Key adipose-related mediators.

**Hormone**	**Major secretion site**	**Cell type**	**Stimulus**	**Receptor**	**Site of action**	**Main effects**	**Impact in BE/EAC**
Leptin	White adipose Stomach	AdipocytesP-cells	Insulin Insulin, gastrin, secretin *via* gastric vagal fibers	Ob-R	Hypothalamus Immune cells Vagal afferents I-cells	Adipostasis, possible post-prandial satiety hormone ↓ *Energy intake* ↑ *Energy expenditure* ↑ *Sympathetic tone* ↓ *Glucagon* ↑ Central sensitivity to post-prandial satiety hormonesAdipokine effect	Adipokine *↑ Systemic inflammation* *↑ T-helper 1* *↑ TNF-α IL-2 IL-6* Direct effect on esophageal mucosa *↑ Proliferation* *↑ Invasiveness* *↑ Migration* *↓ Apoptosis*
Insulin	Pancreas	β-cells	Circulating nutrients *Glucose Amino acids (e.g., l-arginine via GPRC6A) Fatty acids (e.g., ethanolamides via GPR119, MCFA/LCFA via FFAR1)*	Insulin receptor	Adipose Skeletal muscle Hepatocyte Hypothalamus Esophagus	Anabolism *↑ Glucose uptake in skeletal muscle and liver* ↑ *Glycolysis* ↑ *Glycogen synthesis* ↓ *Glycogenolysis* ↓ *Gluconeogenesis* ↑ *Fatty acid synthesis* ↑ *Triglyceride synthesis* ↓ *Lipolysis* ↓ *Proteolysis* ↓ Energy intake	↑ Leptin ↓ Adiponectin ↓ IGF binding protein 1 and 2 ↑ IGF-1 *↑ Mitosis* *↓ Apoptosis* *↑ Angiogenesis*
Adiponectin	White adipose Placenta	Adipocytes	Genetic and lifestyle factors, reduced in obesity	AdipoR1AdipoR2T-cadherin - CDH13	Skeletal muscle Liver Adipose Central nervous system	↑ Insulin sensitivity ↓ *Gluconeogenesis* ↑ *Glucose uptake in skeletal muscle and liver* ↑ Lipolysis ↓ Inflammation ↑ Energy expenditure ↓ Energy intake	Reduced systemic inflammation *↓ TNF-α* Opposes leptin induced carcinogenesis *↓ JAK2 activation* *↓ STAT3 activity*

*FFAR, free fatty acid receptor; GPR, G protein-coupled receptor; IL, interleukin; LCFA, long chain fatty acids; MCFA, medium change fatty acids; TNF-α, tumor necrosis factor alpha*.

Overall, leptin is a proinflammatory mediator, activating proinflammatory cells, stimulating the T-helper 1 cell response and production of IL-2, IL-6, and TNF-α, contributing to the systemic inflammatory milieu in obesity (89). In addition to its function as a proinflammatory cytokine, leptin appears to act directly on the esophageal epithelium. BE and EAC cells express the leptin receptor at high levels (90, 91), while *in vitro* studies demonstrate increased proliferation, invasiveness and migration, and reduced programmed cell death among EAC cells treated with leptin (92). Upregulated leptin receptor expression is associated with anthropometric and radiological measures of obesity among patients with EAC, and with advanced tumor and nodal stage (93). GERD-independent associations between circulating leptin concentrations and the incidence of BE have been observed in a number of studies (94, 95), highlighting the role of adipose inflammation in the development of BE in obesity.

Adiponectin is a complement-like protein secreted by adipocytes which exists in the circulation at levels inversely proportionate to total body fat, and functions to increase insulin sensitivity, and well as exerting anti-inflammatory effects, acting in counterbalance to leptin (91, 92, 96, 97). Adiponectin appears to inhibit leptin-induced carcinogenesis (92), reducing JAK2 activation and *STAT3* activity (75, 92). In addition, adiponectin receptors are expressed in BE and EAC (90, 91), with increased expression associated with less advanced disease stage and improved overall survival (91).

### Insulin and Insulin-Like Growth Factor 1

Insulin circulates at concentrations proportionate to total body fat, as a result of increasing peripheral insulin resistance with increased fat mass (98). Insulin resistance in obesity occurs due to excess circulating FFAs which produce a shift in hepatic glucose metabolism, with reduced gluconeogenesis and glycolysis, and increased lipid oxidation and storage. In obesity, high circulating levels of insulin result in upregulation of insulin receptor expression and associated changes in intracellular signaling pathways which together produce impaired insulin sensitivity in adipose, liver, and muscle (99, 100). Adipokines and circulating reactive oxygen species (ROS) also increase insulin resistance, reducing utilization of ingested nutrients and resulting in high circulating levels of glucose and FFAs. This further increases hepatic insulin resistance, increasing systemic inflammation *via* activation of ROS and increases in IL-6, MCP-1, and TNF-α, and reductions in adiponectin, in a self-perpetuating disease cycle (100, 101).

Hyperinsulinemia and insulin resistance increase the risk of BE among individuals without diabetes, an effect which is mediated at least in part by leptin, and hence due to increased adiposity (95). The association between type T2DM and EAC and BE is inconsistent however (102), as BE risk does not correlate with serum insulin levels among patients with established T2DM (95, 103). This may reflect uncoupling of peripheral insulin resistance and insulin secretion among patients with advanced metabolic disease, reflective of β-cell dysfunction among patients with severe or longstanding T2DM, and suggests that insulin itself may not be direct driver of the inflammation-metaplasia-dysplasia sequence in EAC. However, insulin may exert a pro-tumorigenic effect through a number of downstream pathways. First, insulin promotes leptin secretion and reduces adiponectin expression. Second, hyperinsulinemia reduces insulin-like growth factor (IGF) binding protein 1 and 2, which usually binds and inactivates IGF-1, thereby increasing free IGF-1 which is increased in VO. IGF-1 is increased in EAC compared with BE and healthy control subjects (104, 105). Activation of the IGF-1 receptor results in downstream signaling that increases mitogenic, anti-apoptotic and proangiogenic factors, which may result in tumorigenesis in EAC (96, 105, 106). In *ex vivo* models, patients with VO exhibit increased tumor IGF-1 receptor expression, and this has been linked to reductions in disease-specific survival (104). Together these findings suggest that insulin resistance may increase the risk of EAC through increased systemic inflammation, establishing a self-perpetuating cycle of worsening metabolic dysregulation, and through IGF-1 signaling, which may directly influence carcinogenesis through mitogenic and anti-apoptotic effects and promotion of angiogenesis. Interestingly, recent data from genome-wide association studies (GWAS) indicate that genetic variation in the IGF pathway, specifically cell surface receptors GHR and IGF1R, may influence the risk of BE (107).

### Sex Steroids

EAC is markedly more common in males, with a male: female ratio of 9:1. Sex steroids exhibit an established direct role in the pathogenesis of breast and endometrial cancer, however the impact of sex steroids in EAC has been less well-characterized to date (108, 109). One previous population study demonstrated an association between circulating testosterone and DHT and BE risk among male subjects, while estrone sulfate levels were shown to be protective (110). However, a large study showed no association between plasma hormone concentrations and BE or EAC, but did observe increased risk of BE in association with estrogens in younger men, and with free androgens among men with higher waist-to-hip ratios (111). The latter findings may be more consistent with the role of sex-steroid associated changes in body composition as a risk factor for EAC.

The distribution of adipose tissue differs markedly between males and females, and is regulated by sex steroids, particularly estrogens, which may account in part for the difference in incidence of esophageal cancer between males and females (112, 113). Estrogens have been shown to impact adipocyte differentiation, and also have key roles in the regulation of insulin resistance and lipid metabolism. In males, adipose deposition occurs preferentially in visceral compartment, while females exhibit greater subcutaneous adipose tissue stores (114), however we now know that the VAT in males also exhibits a markedly different immunological landscape as compared with females. Male VAT exhibits increased inflammation, and sex hormones have been shown to regulate VAT inflammation, and T-cell differentiation within the VAT, with potential implications for obesity associated carcinogenesis (115). As such, the increased incidence of EAC in males may be related to increased risk factors among males, including sex-steroid associated increases in VO and adipose inflammation.

## Novel Vistas

### Tumor Microenvironment

A current focus of much research in EAC is the impact of obesity on the tumor microenvironment (TME), the cellular environment in which a tumor exists. The TME includes the extracellular matrix, fibroblasts, immune and inflammatory cells, signaling molecules, and tumor-related blood vessels. A number of processes are dysregulated both in VAT and within the TME, highlighting potential links between obesity and cancer, including hypoxia, inflammation, angiogenesis, energy metabolism, and epithelial to mesenchymal transition (EMT) (20). Obesity may impact the TME both locally, and systemically *via* circulating adipokines, growth factors, and endocrine signals associated with VAT inflammation. The abundance of adipocyte stem cells is increased among individuals with obesity. In murine models, cellular migration to tumor sites has been observed, with differentiation into key cell types know to affect the TME. Adipocyte stem cells may represent a source of cancer-associated fibroblasts (CAFs), with interstitial fibrosis within the TME altering cytokine signaling, and epithelial morphology and differentiation (19, 20, 73). In the TME, obesity and inflammation promote metabolic reprogramming and angiogenesis. Obesity may also disrupt the cancer immunity cycle (116). This model describes the fundamental antitumor immune response, beginning with antigen presentation by maturing dendritic cells on MHC Class I or II molecules to naïve T cells in tumor associated lymph nodes, leading to T cell activation and clonal expansion. CD8+ cytotoxic T cells will then recognize and kill their target cells, resulting in release of tumor antigens propagating the dendritic cell response. Obesity may enable tumors to circumvent immunosurveillance, through hypoxia, acidosis, and nutrient deprivation. Further understanding of how obesity impacts the immune landscape within the TME to impair antitumor immunity may offer novel treatment approaches for EAC (117).

### The Microbiome

There is compelling evidence that the gut microbiome plays a central role in the regulation of inflammation and metabolism, with recent data implicating dysbiosis in the pathophysiology of a number of inflammatory and metabolic conditions, such as inflammatory bowel disease, obesity and MetS. Interestingly, the impact of dietary factors with respect to the development of obesity and MetS may be mediated by host-microbiota interactions, as germ-free mice do not develop metabolic dysregulation in response to a high fat diet (HFD), however fecal transplant to HFD-fed germ-free mice from obese mice increases recipient adiposity (118). Further evidence for the role of microbiota with respect to metabolic status derives from fecal transplant studies, wherein transplant of enteric content from rodents with weight loss post gastric bypass surgery to sham operated controls produced significant weight loss (119). Thus, it appears the gut microbiome plays a key role in the regulation of metabolism, and therefore its impact on carcinogenesis in obesity is the focus of increasing research.

Obesity, HFD, and GERD may alter the composition of the esophageal microenvironment, resulting in changes in the resident microbiota. Early data indicate that individuals with GERD and BE exhibit distinct patterns of microbiota (120, 121), with Gram-positive bacteria from the Firmicutes phylum most abundant in the healthy esophagus, whereas increasing proportions of Gram-negative anaerobes and microaerophiles of the phyla Bacteroides, Proteobacteria and Fusobacteria (termed a “type II” microbiome) are seen among patients with GERD and BE (122). Fusobacteria have been shown to contribute to the pathophysiology of colorectal cancer, through promotion of the local inflammatory response. Rodent models examining the impact of dietary modification now indicate that metabolic parameters are strongly correlated with esophageal microbiota signatures, and host esophageal gene expression. A number of phyla including Fusobacterium, Rothia, and Granulicatella showed consistent relationships across a range of metabolic and gene markers, indicating that HFD can significantly alter the esophageal microbiota, enriching bacterial species previously shown to be associated with gastrointestinal carcinogenesis (123). Further research is urgently required to better understand the impact of shifts in the complex microbial ecology of the upper gastrointestinal tract on metabolic health and carcinogenesis.

### Common Genetic Risk

A considerable body of literature now supports the overall link between obesity and several cancers, and a number of studies have assessed whether this association may be explained in part by common genetic variants (124–129). A prototypical example is rs294364. This single nucleotide polymorphism (SNP) is associated with the insulin receptor substrate 1 (*IRS1*) gene and confers improved insulin sensitivity, fat distribution and metabolism. Variants in *IRS1* were recently assessed among participants in the Swedish Obese Subjects (SOS) study (130), a large case-control study which has yielded major insights regarding long-term outcomes following bariatric surgery (131–134). Variants in *IRS1* were associated with improved insulin resistance, and a lower incidence of cancer among patients treated with usual care, but not those who underwent bariatric surgery. The incidence of specific cancer types was not specifically reported, and therefore generalizability to EAC remains uncertain.

GWAS demonstrate that ~35% of the risk of BE may be explained by genetic variants, with a high genetic correlation and polygenic overlap between BE and EAC, indicating that common genetic pathways underlie the development of BE and EAC (135). Early GWAS also demonstrated that variants in genes involved in esophageal development, such as *FOXF1*, are associated with increased risk of BE, in addition to variants within the major histocompatibility complex locus on the short arm of chromosome 6. Furthermore, for 29 of 40 SNPs associated with obesity, the same allele increased risk of BE (136). These findings have since been validated, with the association with variants in *FOXF1* now shown to be associated with EAC (137, 138). Additional genes involved in esophageal development such as *BARX1, TBX5*, and *FOXP1* (137, 139, 140), and genes implicated in inflammatory and oncogenic pathways such as *CRTC1* and *GDF7* have also been identified as associated with increased risk of BE and/or EAC (137, 139–141).

An SNP near the *EIF2C3* has recently been reported to interact with BMI to increase risk of BE (142). This gene encodes a protein required for RNA-mediated gene silencing which modulates short RNAs, such as microRNAs (miRNAs) or short interfering RNAs and represses the translation of mRNAs complementary to them. Similarly, several SNPs associated with miRNAs, non-coding RNAs implicated in post-transcriptional gene regulation which may be dysregulated in EAC, have been linked to increased risk of BE and/or EAC. These included miRNA biogenesis genes, gene loci, and miRNA targeted mRNAs, however no interaction with genes implicated in obesity was identified (143). These data provide evidence that multiple SNPs with small effect size additively impact BE risk, and that common genetic variants may underlie the observed epidemiologic association between obesity, BE and EAC.

## Impact of Obesity on Cancer Treatment Outcomes

### Chemotherapy

Obesity may be associated with increased risk of chemotherapy toxicity among patients with EAC. This is particularly pronounced among patients with sarcopenic obesity, a condition characterized by elevated BMI but reduced lean body mass or skeletal muscle index on body composition assessment (144, 145). For example, one study demonstrated a 5.5-fold increased risk for dose-limiting chemotoxicity among patients with sarcopenic obesity, with no increased risk among normal-weight patients with sarcopenia (145). Increased risk of dose-limiting toxicity may be due to reduced volume of distribution in lean body mass relative to calculated body surface area, with significant variation in the effective volume of distribution in obesity (145, 146). In addition, patients with obesity often exhibit a greater number of baseline comorbidities, which may impact treatment tolerance (147, 148). In some studies, patients with obesity were less likely to receive neoadjuvant therapy despite a similar stage at presentation (149). Future research may assess the role of lean body mass-based, vs. conventional body surface area (weight) based, calculations for chemotherapy dosing (for example NCT01624051), to reduce treatment toxicity, particularly among patients with sarcopenic obesity.

### Surgery

Intuitively, obesity may increase the technical complexity of major oncologic surgery, and metabolic dysfunction may introduce added perioperative risk factors. Esophageal cancer surgery is associated with significant morbidity, and anastomotic leak, pneumonia, and atrial fibrillation are the most common major complications. Patients with VO and/or MetS may have greater respiratory and cardiac comorbidity, and T2DM, and reduced functional capacity, in particular with respect to pulmonary physiology (147, 148). Baseline lung function testing predicts postoperative pulmonary complications among patients undergoing esophagectomy. Meta-analysis shows that all measures of lung function are decreased among subjects with obesity (150). A reported obesity paradox exists in pneumonia unrelated to surgery, with an increased risk yet reduced or unchanged mortality, and whether a similar effect may be observed among patients with obesity undergoing surgery for EAC requires further study (151).

While overall postoperative morbidity rates appear to be similar among patients with obesity compared with normal weight and underweight individuals, specific complications may be increased among patients with obesity. A recent meta-analysis reported a markedly increased risk of anastomotic leak, up to 35%, among patients with BMI-defined obesity, but otherwise similar postoperative outcomes (26). A clear technical difficulty may be the preparation of an optimal gastric conduit, without tension. In addition, factors such as atherosclerosis and diabetes may increase the risk of ischemia, particularly when compounded by impaired pulmonary oxygen exchange (150). It remains to be determined whether severe obesity, or obesity associated with MetS, is associated with greater postoperative morbidity, as compared with normal weight or mild obesity. Of note, VO and MetS have been shown to be associated with an exaggerated postoperative inflammatory response (152, 153).

### Survival

Despite differences in tolerance to preoperative (neoadjuvant) chemotherapy or chemoradiotherapy in esophageal cancer, limited data suggest that obesity is associated with similar response rates following neoadjuvant therapy (147, 154). In comparative research using BMI as the measure of obesity, several studies demonstrate that increased BMI is associated with improved survival outcomes (148, 154, 155), with meta-analysis showing significantly (RR = 1.17 [95% CI: 1.03–1.32]) better survival among patients with obesity (26). Sarcopenic obesity however be associated with adverse survival outcomes among patients with esophageal cancer (156). For VA specifically, a solitary study of 126 patients assessed its impact on survival among patients with EAC and reported no impact of visceral obesity above a median, but worsened outcomes with increasing visceral obesity presented in quartiles, principally due to more advanced clinical stage (157).

Insulin resistance, IGF-1, leptin, and IL-6 may be increased among patients with MetS, and may negatively impact outcomes for cancer as for cardiovascular diseases (158, 159). Meta-analysis of survival outcomes according to the presence of MetS among patients with gastrointestinal cancers demonstrated a significant increase in mortality risk among patients with MetS in prospective studies (HR = 1.64 [95% CI: 1.18–2.28]), studies involving postsurgical patients (HR = 1.42 [95% CI: 1.06–1.92]) and those assessing cancer-specific survival (HR = 1.91 [95% CI: 1.45–2.52]) (160). However, no previous studies have assessed the impact of MetS on survival outcomes among patients with EAC.

## Risk Modification

### Diet and Weight Loss

Mouse studies demonstrate that HFD is associated with the development of esophageal dysplasia and adenocarcinoma, associated with increased IL-8 and a shift in gut microbiota (161). Despite this, few studies have examined the role of dietary intervention for prevention of EAC or progression of BE to EAC (162, 163). Observational data from the Netherlands Cohort Study demonstrated that a Mediterranean diet was not associated with reduced risk of EAC (164). The Look AHEAD randomized controlled trial assessed the role of intensive lifestyle intervention vs. conventional care with a primary outcome measure of cardiovascular events. This study did not show a reduction in cardiovascular events and was closed early after 10 years, despite weight loss of 8.6% compared with 0.7% at 1 year and 6% compared with 3.5%, in the intervention and control groups, respectively. However, follow-up of cancer incidence between intervention and control groups is eagerly anticipated (165). Future studies may advance our knowledge by utilizing prospective diaries and validated dietary questionnaires and recording of key confounders such as GERD and obesity.

Bariatric/Metabolic surgery is a highly effective intervention for the treatment of obesity, resulting in sustained weight loss and improvements in aspects of the MetS such as insulin resistance and altered lipid metabolism (166). Approximately 70% of patients with T2DM experience remission after Roux-en-Y gastric bypass (RYGB) and sleeve gastrectomy (SG), with reductions in cardiovascular, all cause and cancer mortality among patients with obesity who undergo bariatric surgery, compared with best medical therapy (166–168). Patients who underwent bariatric surgery in the Swedish Obese Subjects study demonstrated reduced incidence of cancer (HR = 0.67 [95% CI: 0.53–0.85]) in long-term follow-up (169). Although this effect appeared to be driven by reduced cancer incidence in women, the young age of patients in the study and the relatively higher overall incidence of obesity associated cancers in women (namely breast and endometrial cancer) may have reduced the sensitivity of the study to detect an effect for EAC. However, the significant weight loss and improvements in glycaemia, reductions in circulating insulin and improved systemic inflammatory milieu observed after bariatric surgery may result in reduced EAC risk (170, 171). Additional factors such as reduced acid and bile reflux, particularly after Roux-en-Y gastric bypass, may be associated with reduced BE and EAC risk (172–174). Future studies may delineate the interplay of factors impact BE and EAC risk among patients following bariatric surgery, to aid procedure selection among patients with baseline GERD.

### Physical Activity

Reduced physical activity has been found to be associated with increased risk of EAC in multiple studies (175, 176). A large meta-analysis has demonstrated reduced EAC risk among patients with greater physical activity, with evidence of a dose response effect (177). As such, lifestyle interventions comprising increases in physical activity may be useful as part of the primary prevention of EAC among at-risk individuals. In this regard, the ongoing Exercise and the Prevention of Esophageal Cancer (EPOC) study represents a randomized controlled trial including a mixed aerobic and resistance exercise intervention among men with BE who have obesity and live a sedentary lifestyle at baseline. The primary outcome measure will be progression of BE, with secondary outcome measures encompassing a comprehensive metabolic assessment, GERD severity and indices of cardiovascular fitness and strength (178). This trial will provide empiric evidence to support exercise advice for individuals with BE and will clarify whether the observed link between reduced physical activity and EAC represents a causal relationship.

### Proton Pump Inhibitors

A previous multicenter prospective cohort study demonstrated reduced risk of BE progression among patients treated with proton pump inhibitors (PPIs) over a median 5.2 year follow-up period (179). A systematic review and Delphi consensus found no strong evidence to support the use of PPIs or low-dose aspirin for chemoprophylaxis in BE (180). Subsequent to this the recent ASPECT trial was conducted at 84 centers across the United Kingdom and Canada, and utilized a 2 × 2 factorial design to assess the impact of high dose vs. low dose PPIs, with or without aspirin, to assess a composite endpoint of all-cause mortality, EAC or HGD among patients with BE. High dose PPI was superior to low dose PPI (139 events in 1,270 patients vs. 174 events in 1,265 patients), with a number needed to treat (NNT) of 34 for high dose PPI and a low rate of adverse events (181). These data suggest that high dose PPIs are effective in the prevention of EAC, HGD, and mortality among patients with BE providing high level evidence to support their routine use. The impact of obesity, in particular VO on this response, requires further study.

### Aspirin

Given the role of systemic inflammation in the pathogenesis of BE and EAC, a key question is whether modulation of the inflammatory response may prevent BE or its progression (182). Observational data indicate a reduced incidence of EAC among patients prescribed non-steroidal anti-inflammatory drugs for another indication (183). This has prompted the study of cyclooxyengase-2 (COX-2), a membrane-bound glycoprotein involved in prostanoid synthesis, in BE-associated carcinogenesis (184–186). Exposure to bile acids, acid and greater adiposity are associated with increased COX-2 expression in esophageal squamous mucosa. Similarly, expression of COX-2 is significantly increased in BE and EAC compared with normal mucosa (187, 188). Increased COX-2 signaling is thought to predispose to carcinogenesis though resistance to apoptosis, angiogenesis and proliferation, and increased invasiveness. Importantly, elevated COX-2 expression has been associated with adverse oncologic outcomes among patients with EAC (189). However, a multicenter phase IIb randomized controlled trial of the COX-2 inhibitor celecoxib demonstrated no difference in the proportion of biopsy samples showing dysplasia or cancer among patients with dysplastic BE at enrolment (190).

Epidemiologic data indicates that aspirin may confer a protective effect with respect to BE/EAC risk (184–186). Aspirin is known to irreversibly inactivate COX-2, resulting in reduced downstream production of prostaglandins and thromboxane A2. The aforementioned ASPECT trial compared the addition of aspirin to high dose or low dose PPI among patients with BE and found that aspirin was not significantly better than no aspirin (127 events in 1,138 patients vs. 154 events in 1,142 patients), however when participants using non-steroidal anti-inflammatory drugs excluded, a significant beneficial effect was observed with aspirin. Combined treatment with high dose PPI and aspirin had the strongest effect, and the NNT for aspirin was 43 to prevent one event (all-cause mortality, EAC or HGD) (181). As for PPIs, the impact of VO and MetS on response requires further study.

### Other Medical Approaches

Metformin has also been suggested as a possible therapeutic to prevent obesity-associated cancers, by attenuating hyperinsulinemia. A randomized controlled study of patients with BE assessed the impact of metformin on levels of phosphorylated S6 kinase (pS6K1), a marker of insulin pathway activation. The study found no significant change in pS6K1, and a trend toward reduced insulin levels and improved insulin resistance, and there was no significant difference in epithelial proliferation or apoptosis in esophageal biopsies. As such current data do not support the use of metformin as chemoprevention among patients with BE (191). Observational population level data have suggested that exposure to statins may be associated with reduced EAC risk (192). A previous meta-analysis including 13 studies demonstrated a significant 28% reduction in EAC among individuals in receipt of statins, with a 41% reduction in EAC risk among patients with an established diagnosis of BE (193). However, the NNT to prevent one case of EAC among patients with BE was 389, and therefore economic analysis concluded that statins would only be appropriate for patients at high risk for BE progression (194).

## Conclusion

EAC is an exemplar of an obesity associated cancer, with the International Agency for Research on Cancer (IARC) recently reporting a progressive increase in relative risk for EAC for each 5 kg/m^2^ increase in BMI, suggesting a dose-response effect (15). Visceral fat is the key adipose compartment linking obesity to carcinogenesis, through increased systemic inflammation, insulin resistance, IGF-1, and immune cell alterations, as well as promoting inflammation in the TME. In addition, obesity and HFD modulate the gut microbiota, enriching bacterial species known to be associated with gastrointestinal carcinogenesis. Obesity, in particular sarcopenic obesity, is associated with increased chemotoxicity among patients with EAC, likely due to reduced effective volume of distribution relative to weight-based dosing calculations. Postoperatively, obesity is associated with increased risk of anastomotic leak, but other complications appear to occur with similar frequency. The postoperative systemic inflammatory response is increased among patients with VO; however, few studies have assessed the impact of VO and MetS on postoperative outcomes. Among patients with EAC, meta-analysis supports the presence of an obesity paradox, whereby obesity is associated with increased risk for development of EAC overall, but is also linked to favorable survival outcomes. Mechanisms underlying this effect are uncertain, and future work may delineate whether this represents confounding by weight loss at diagnosis, or altered tumor biology among patients with obesity-associated esophageal carcinogenesis. Despite a significant body of evidence supporting the link between VO, MetS, and BE and EAC risk, the role of dietary, lifestyle and bariatric surgical interventions, and of pharmacologic and pharmabiotic therapies, to modulate the risk of EAC remains unclear and requires further clinical and translational study.

## Author Contributions

JE and JR designed and conducted the research, provided essential materials, analyzed data, wrote the paper, and have primary responsibility for final content. All authors contributed to the article and approved the submitted version.

## Conflict of Interest

The authors declare that the research was conducted in the absence of any commercial or financial relationships that could be construed as a potential conflict of interest.
